# 

**Published:** 1877-02

**Authors:** 


All Subscriptions and Communications to this Journal should be sent to the EDITOR, at the office of publication, 115 State Street.

VOL. XXXIV.
NUMBER 2.

February, 1877.

NEW SERIES,
VOL. II.
NUMBER 2.

### THE CHICAGO MEDICAL Journal and Examiner

PUBLISHED MONTHLY.

**EDITOR:**
WILLIAM H. BYFORD, A.M., M.D.

**ASSOCIATE EDITORS:**

JAMES H. ETHERIDGE, M.D. | JAS. NEVINS HYDE, A.M., M.D.

NORMAN BRIDGE, M.D. | FERD. C. HOTZ, M.D.

Published under the auspices of the

*CHICAGO MEDICAL PRESS ASSOCIATION.*

**TERMS—FOUR DOLLARS PER ANNUM, IN ADVANCE.**
**POSTAGE FREE.**

S.G.O.

CHICAGO:

PUBLISHED BY

CHICAGO MEDICAL PRESS ASSOCIATION,

NOS. 113 AND 115 STATE STREET.

☞ Our Patrons and Contributors will kindly assist The Chicago Medical [illegible]

